# Biological Evaluation and Docking Analysis of Potent BACE1 Inhibitors from *Boesenbergia rotunda*

**DOI:** 10.3390/nu11030662

**Published:** 2019-03-19

**Authors:** Kumju Youn, Mira Jun

**Affiliations:** 1 Department of Food Science and Nutrition, College of Health Sciences, Dong-A University, 37, Nakdong-daero 550 beon-gil, Saha-gu, Busan 49315, Korea; kjyoun@dau.ac.kr; 2 Center for Silver-Targeted Biomaterials, Brain Busan 21 Plus Program, Graduate School, Dong-A University, Nakdong-daero 550 beon-gil, Saha-gu, Busan 49315, Korea; 3 Institute of Convergence Bio-Health, Dong-A University, Busan 49315, Korea

**Keywords:** Alzheimer’s disease, Aβ, BACE1, in silico docking, *Boesenbergia rotunda*

## Abstract

Alzheimer’s disease (AD) is an irreversible neurodegenerative disorder characterized by progressive impairment of cognitive functions. Beta-site amyloid precursor protein cleaving enzyme1 (BACE1) is essential for the formation of β-amyloid peptide (Aβ), a major constituent of amyloid plaques that represent a neuropathological hallmark of this disorder. To find alternative therapies for AD sourced from natural products, the present study focused on three flavonoids from *Boesenbergia rotunda*, namely, cardamonin, pinocembrin, and pinostrobin. Biological evaluation showed that cardamonin presented the strongest BACE1 inhibition, with an The half maximal inhibitory concentration (IC_50_) value of 4.35 ± 0.38 µM, followed by pinocembrin and pinostrobin with 27.01 ± 2.12 and 28.44 ± 1.96 µM, respectively. Kinetic studies indicated that the inhibitory constants (*K_i_*) for cardamonin, pinocembrin, and pinostrobin against BACE1 were 5.1, 29.3, and 30.9 µM, respectively. Molecular docking studies showed that the tested compounds did not bind to the BACE1 active site, consistent with the biological results, illustrating non-competitive inhibitory activity for all three compounds. In addition, the lowest binding energy of the most proposed complexes of cardamonin, pinocembrin, and pinostrobin with BACE1 were −9.5, −7.9, and −7.6 kcal/mol, respectively. Overall, we provide the first evidence that these flavonoids from *B. rotunda* may be considered as promising AD preventative agents through inhibition of Aβ formation.

## 1. Introduction

Alzheimer’s disease (AD) is an irreversible neurodegenerative disorder that results in progressive behavioral and cognitive impairment. The pathological hallmarks of AD are extracellular beta-amyloid (Aβ) plaque deposits and intracellular neurofibrillary tangles (NFTs) of the microtubule-binding protein tau [1]. Numerous studies strongly support a major role for Aβ aggregates in the initiation phase of AD pathogenesis, although the precise mechanisms of Aβ-induced neurotoxicity remain unclear. Aβ is derived from sequential proteolytic cleavage of the amyloid precursor protein (APP) by two aspartic proteases, β- and γ–secretase. β-Secretase (BACE1) initiates amyloidogenic APP processing, generating sAPPβ and CTF99, and resulting in the processing of CTF99 in the transmembrane region by the γ-secretase complex and then produces the Aβ_40_ and Aβ_42_ peptides [2,3,4]. Increasing evidence suggests that Aβ generated by BACE1 triggers neuroinflammatory responses by inducing the expression of inflammatory cytokines and mediators via nuclear factor-κB (NF-κB) activation [5]. In addition, reactive oxygen species (ROS) have been revealed to cause the formation of Aβ fibrils, which in turn accelerates oxidative stress, inflammatory responses, and more Aβ accumulation, ultimately leading to cell death. Recently, it was reported that BACE1 directly contributed to inflammation, indicating that the targeting BACE1 provides suppression of inflammatory responses [6].

Drug discovery efforts aimed at preventing the production of Aβ have focused on targeting the enzymes involved in the amyloidogenic processing of APP. Although several γ-secretase inhibitors have been indicated to inhibit Aβ generation, γ-secretase has a physiologically essential substrate, the Notch signaling protein, which plays a crucial role in the differentiation and proliferation of many cell types [7]. In contrast, BACE1 is a promising alternative for the treatment and/or prevention of AD, considering the relatively mild phenotypes revealed in BACE1-null mice [8,9]. The first generation of BACE1 inhibitors consisted of large peptide-based transition-state analogues, which showed poor in vivo pharmacological properties including a large molecular weight, oral biounavailability, short serum half-life, or low blood–brain barrier (BBB) penetrance [10]. Subsequently, new classes of non-peptidic, lipophilic, small molecule BACE1 inhibitors were designed with improved pharmacological properties. However, these second-generation BACE1 inhibitors were unable to achieve sufficiently high concentrations in the brain due to poor BBB penetration [11]. In contrast, third-generation orally bioavailable BACE1 inhibitors have elicited reduced Aβ production in preclinical animal trials, and some have even entered human clinical trials [12]. The results of several clinical trials showed cerebral Aβ reduction with once-daily oral dosing, although off-target effects, including liver toxicity and skin depigmentation, were also observed [13].

*Boesenbergia rotunda* (L.) Mansf., also known as fingerroot and Chinese ginger, is widely found in Asian countries including India, Sri Lanka, and southern China. It is commonly used as a food ingredient, as well as in traditional medicine to treat tumors, diarrhea, swelling, and dermatitis [14]. Bioactive *B. rotunda* compounds have been categorized into two major groups, flavanones and chalcones [15]. The main flavanones, like pinocembrin and pinostrobin, and a major chalcone, cardamonin, act as anti-oxidant, anti-inflammatory, anti-tumor, and anti-tuberculosis agents [16]. In addition, these compounds were reported to promote neuroprotective effects against Aβ, glutamate, oxidative stress, inflammation, and ischemic injury in several in vitro and in vivo studies [17,18,19,20,21,22,23,24,25,26]. According to the previous research, pinocembrin attenuated 6-OHDA- and Aβ-induced neuronal cell death through the Nrf pathway in SH-SY5Y cells [21,22]. Oral administration of pinocembrin (10 mg/kg) for 7 days suppressed hippocampal inflammation, oxidative stress, and apoptosis in a rat model of global cerebral ischemia-reperfusion [23]. Liu et al. reported that when pinocembrin was orally administered at 20 mg/kg for 8 days, it significantly improved cognitive impairment in intracerebroventricular Aβ_25–35_-injected mice [24]. Moreover, chronic administration of this compound (40 mg/kg) for a 3-month period attenuated cognitive deficits and protected the neurovascular unit in an APP/PS1 transgenic mouse model [25]. Pinostrobin, meanwhile, significantly reduced oxidative stress and mitochondrial-mediated neural apoptosis against Aβ-induced neurotoxicity in PC12 cells [15]. Recently, pinostrobin was also reported to improve neuronal cell loss and deficient locomotive behavior induced by 1-methyl-4-phenyl-1,2,3,6-tetrahydropyridine (MPTP) in zebrafish, and to protect SH-SY6Y cells against 1-methyl-4-phenylpyridinium (MPP+)-induced oxidative stress and apoptosis, suggesting that pinostrobin has neuroprotective potential both in vitro and in vivo [26]. Although cardamonin is the least studied of the three flavanones, it was shown to afford neuroprotection against oxidative injury in PC12 cells and to inhibit neuroinflammation in the mouse microglia BV2 cell line [18,19].

The three compounds from *B. rotunda* were proven to possess anti-inflammatory properties in neuronal cell models, suggesting that these three compounds might regulate inflammatory response by way of suppressing BACE1 activation. Therefore, in the present study, for the first time, the direct effects of these compounds against BACE1 were examined through in vitro and in silico docking approaches.

## 2. Materials and Methods

### 2.1. Reagents

Cardamonin (>98%), pinocembrin (>95%), pinostrobin (>99%), trans-resveratrol (>99%), chymotrypsin, trypsin, elastase, and their substrates were purchased from Sigma-Aldrich (St. Louis, MO, USA). The BACE1 assay kit was purchased from Invitrogen (Pan Vera, Madison, WI, USA). Tumor necrosis factor- α converting enzyme (TACE), a major α-secretase, and its substrate were bought from R&D Systems (Minneapolis, MN, USA).

### 2.2. In Vitro Enzyme Inhibitory Assay for Biological Evaluation

Enzyme assays for BACE1, TACE, chymotrypsin, trypsin, and elastase were carried out according to previously described methods [27]. Briefly, reaction mixtures containing human recombinant BACE1 (1.0 U/mL), a specific substrate (Rh-EVNLDAEFK-Quencher in 50 mM ammonium bicarbonate), and samples dissolved in an assay buffer (50 mM sodium acetate, pH 4.5) were incubated for 60 min at 25 °C in well plates. The fluorescence intensity produced by substrate hydrolysis was observed on a microplate reader with excitation and emission wavelengths of 545 and 590 nm, respectively. The inhibition ratio was obtained using the following equation:

Inhibition (%) = [1 − (S − S_0_)/(C − C_0_)] × 100

where C was the fluorescence of control (enzyme, assay buffer, and substrate) after 60 min of incubation, C_0_ was the fluorescence of control at time 0, S was the fluorescence of tested samples (enzyme, sample solution, and substrate) after 60 min of incubation, and S_0_ was the fluorescence of the tested samples at time 0.

A human recombinant TACE (0.1 ppm in 25 mM Tris buffer), the substrate (APP peptide YEVHHQKLV using EDANS/DABCYL), and samples were dissolved in an assay buffer, which were then combined and incubated for 60 min in the dark at 25 °C. The increase in fluorescence intensity produced by substrate hydrolysis was measured by using a microplate reader with excitation and emission wavelengths of 320 and 405 nm, respectively.

Absorbance assays for trypsin, chymotrypsin, and elastase were estimated using N-benzoyl-ʟ-Arg-pNA, N-benzoyl-ʟ-Tyr-pNA, and N-succinyl-Ala-Ala-Ala-pNA as substrates, respectively. Enzyme, Tris-HCl buffer (0.05 M, in 0.02 M CaCl_2_, pH 8.2), and tested samples were incubated for 10 min at 25 °C then added to the substrate for 30 min at 37 °C. The absorbance was recorded at 410 nm. The inhibition ratio was obtained using the following equation:

Inhibition (%) = {[1 − (A − B)]/control} × 100

where A was the absorbance of control (enzyme, assay buffer, and substrate) after 60 min of incubation, and B was the absorbance of the tested sample (assay buffer and sample solution) after 60 min of incubation.

### 2.3. BACE1 Inhibition Kinetics

To determine the kinetic mechanisms of cardamonin, pinocembrin, and pinostrobin, Dixon and Lineweaver–Burk plots were used with varying concentrations of substrate (250, 500, and 750 nM) and inhibitors (0.1, 1, 5, 10, and 25 µM for cardamonin and 1, 10, 25, 37.5, and 50 µM for pinocembrin and pinostrobin). The dissociation constant (*Ki*) was obtained by Dixon plot and maximum reaction velocity (V_max_) and Michaelis–Menten constant (K_m_) values were determined by Lineweaver–Burk plot using SigmaPlot™ version 12.3 (Systat Software, Inc., San Jose, CA, USA).

### 2.4. In Silico Docking Studies

Molecular docking analysis of the interaction between BACE1 and our tested compounds was performed using the AutoDock Vina program to display the binding conformations of the compounds in the enzyme [28]. The amino acid sequence of BACE1 and P-glycoprotein (P-gp) (PDB, 2WJO and 3G60) was acquired from the Protein Data Bank (http://www.rcsb.org). The grid dimension was set to 30 Å × 30 Å × 30 Å with a cluster radius of 1 Å. The Cα coordinates for every used backbone-binding residue of the protein receptor were used for the center of docking space. Default options were selected for other docking simulations. The atomic coordinates of the ligands were drawn and visualized using Marvin sketch (5.11.4, 2012, ChemAxon, Cambridge, MA, USA). The calculated geometries were ranked in terms of the lowest energy, and the top-ranked poses were selected. The hydrogen bond calculation was evaluated using Chimera 10.0.2.

### 2.5. Statistics

All results were presented as the mean ± standard deviation (SD) of three independent experiments. Significant differences were determined by Duncan′s multiple range tests using Statistical Analysis System (SAS) version 9.3 (Cary, NC, USA).

## 3. Results

### 3.1. Anti-BACE1 Activity of Cardamonin, Pinocembrin, and Pinostrobin

**Figure 1:**
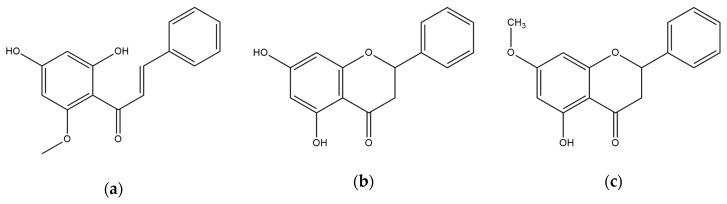
The chemical structures of (**a**) cardamonin, (**b**) pinocembrin, and (**c**) pinostrobin.

The structures of cardamonin (2′,4′-dihydroxy-6′-methoxychalcone), pinocembrin (5,7-dihydroxyflavanone), and pinostrobin (5-hydroxy-7methoxyflavanone) are shown in Figure 1. As depicted in Table 1, cardamonin presented the strongest BACE1 inhibition (IC_50_, 4.35 ± 0.38 µM), followed by pinocembrin (IC_50_, 27.01 ± 2.12 µM) and pinostrobin (IC_50_, 28.44 ± 1.96 µM). In addition, the IC_50_ value for cardamonin was three-fold higher than for resveratrol, which was used as a positive control.

To determine their specificity for BACE1, the compounds were tested on α-secretase (TACE), involved in non-amyloidogenic processes, as well as on other proteases. None of the three compounds significantly inhibited the activity of TACE or serine proteases, including trypsin, chymotrypsin, and elastase, even at a concentration of 100 µM (Table 2).

### 3.2. BACE1 Kinetics of Cardamonin, Pinocembrin, and Pinostrobin

**Figure 2:**
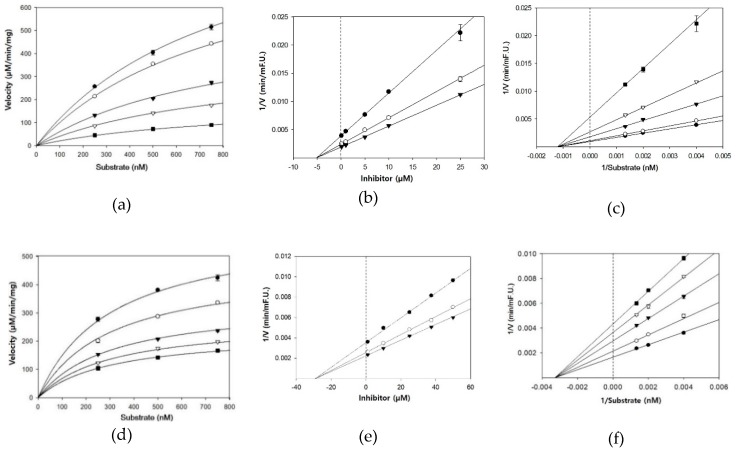

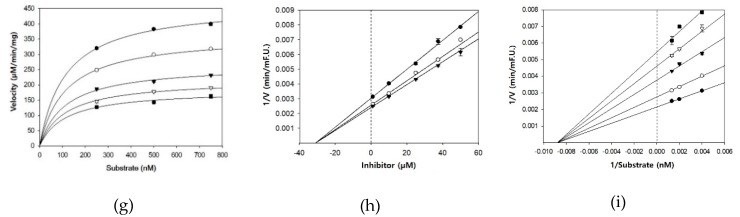
Michaelis–Menten plot of BACE1 inhibition by (**a**) cardamonin, (**d**) pinocembrin, and (**g**) pinostrobin in the presence of different inhibitor concentrations: 0.1 μM (●), 1 μM (○), 5 μM (▼), 10 μM (▽), and 25 μM (■) for cardamonin (a); 1 μM (●), 10 μM (○), 25 μM (▼), 37.5 μM (▽), and 50 μM (■) for pinocembrin (d) and pinostrobin (g). Dixon plot of BACE1 inhibition by (**b**) cardamonin, (**e**) pinocembrin, and (**h**) pinostrobin in the presence of different substrate concentrations: 250 nM (●), 500 nM (○), and 750 nM (▼). Lineweaver–Burk plot of BACE1 inhibition by (**c**) cardamonin, (**f**) pinocembrin, and (**i**) pinostrobin in the presence of different inhibitor concentrations: 0.1 μM (●), 1 μM (○), 5 μM (▼), 10 μM (▽), and 25 μM (■) for cardamonin (c); 1 μM (●), 10 μM (○), 25 μM (▼), 37.5 μM (▽), and 50 μM (■) for pinocembrin (f) and pinostrobin (i).

The type of inhibition and kinetic analysis was determined using a Michaelis–Menten curve, Dixon plot, and Lineweaver–Burk plot (Figure 2). The three lines of the Dixon plots intersected on the x-axis of the same plot (Figure 2b,e,h). In addition, the Lineweaver–Burk plots showed decreased V_max_ and unchanged K_m_ at increasing inhibitor concentrations (Table 1). This pattern indicated non-competitive inhibitory activity against BACE1, thereby interacting with locations other than the active binding site for these compounds. For noncompetitive inhibition of enzymes, the K_i_ value of inhibitor is essentially the same numerical value as the IC_50_ [29]. As shown in Table 1, the K_i_ values for cardamonin, pinocembrin, and pinostrobin were 5.1, 29.3, and 30.9 μM, respectively, which were similar to their IC_50_ values. Because lower K_i_ values represent tighter binding with the enzyme, cardamonin was determined to be the most potent BACE1 inhibitor of the three compounds tested.

### 3.3. Molecular Docking Study of Cardamonin, Pinocembrin, and Pinostrobin with BACE1 and P-Glycoprotein (P-gp)

**Figure 3:**
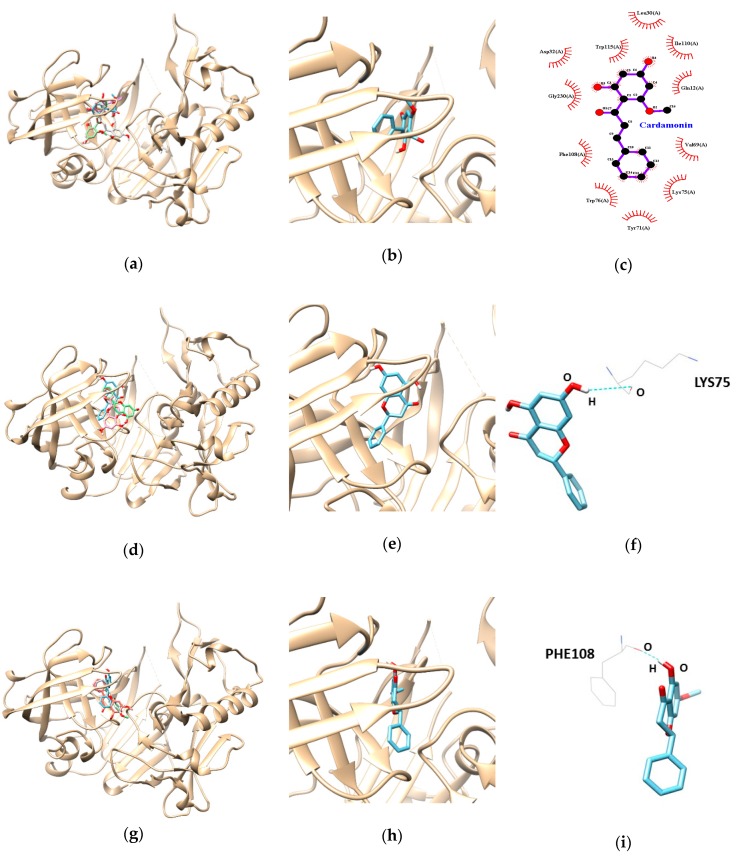
Molecular docking analysis of cardamonin, pinocembrin, and pinostrobin with BACE1. The best docking poses between BACE1 and cardamonin (**a**), pinocembrin (**d**), and pinostrobin (**g**). Magnified view of the binding site for cardamonin (**b**), pinocembrin (**e**), and pinostrobin (**h**). The hydrophobic interaction of cardamonin (**c**). Hydrogen interaction diagram of pinocembrin (**f**), and pinostrobin (**i**). The blue dotted lines show hydrogen bonds between ligands and BACE1.

Based on the biological evaluation results, we performed molecular docking simulations of enzyme-inhibitor conformations and lowest binding energies. As indicated in Table 3, the docking results for cardamonin, pinocembrin, and pinostrobin showed negative binding energies of −9.5, −7.9, and −7.6 kcal/mol, respectively, suggesting that all our three compounds are high-affinity BACE1 inhibitors that can bind tightly to the enzyme. In addition, the pinocembrin-BACE1 complex was stabilized by the formation of a hydrogen bond between residue LYS75 and the hydroxyl group of pinocembrin at a distance of 3.088 Å (Figure 3d–f and Table 3). Pinostrobin is bound at the PHE108 residue of BACE1, connected by one hydrogen bond with a bonding distance of 1.990 Å (Figure 3g–i and Table 3 ). In cardamonin-BACE1 complex, Gln12, Leu30, Asp32, Val69, Tyr71, Lys75, Trp76, Phe108, Ile110, Trp115, and Gly230 of BACE1 participated in hydrophobic interactions with cardamonin without any hydrogen bonding interactions (Figure 3a–c and Table 3).

**Figure 4:**
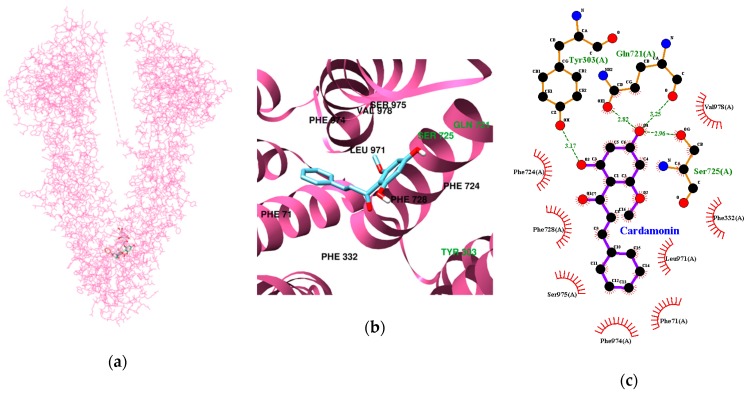

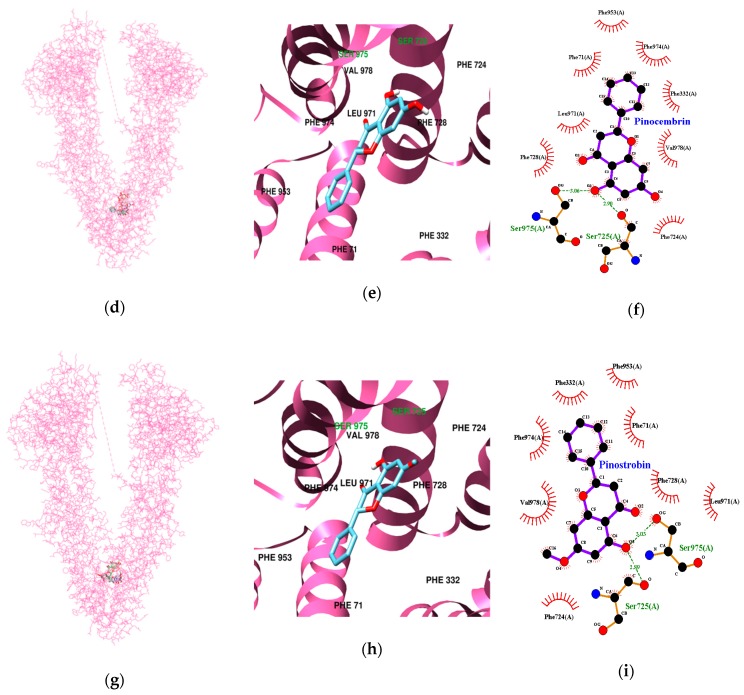
Molecular docking analysis of cardamonin, pinocembrin, and pinostrobin with P-gp. The best docking poses between P-gp and cardamonin (**a**), pinocembrin (**d**), and pinostrobin (**g**). Magnified view of the binding site for cardamonin (**b**), pinocembrin (**e**), and pinostrobin (**h**). Hydrogen and hydrophobic interaction diagram of cardamonin (**c**), pinocembrin (**f**), and pinostrobin (**i**). The green dotted lines show hydrogen bonds between ligands and BACE1.

The multidrug transporter P-glycoprotein (P-gp) is an important component of the BBB, acting as an ATP-driven efflux pump, controlling the movement of structurally diverse molecules across the BBB [30]. To predict the possibility of passing the BBB, the docking simulation between P-gp and our tested compounds was performed and the results are presented in Table 4 and Figure 4. Four hydrogen bonds were found between cardamonin and the P-gp residues, with the lowest binding energy of −9.78 kcal/mol. In addition, Tyr303, Gln721, and Ser725 participated in hydrogen-interactions (bonding distances of 3.17, 2.82, 3.25, and 2.96 Å, respectively). The pinocembrin-P-gp complex (the lowest binding energy: −10.64 kcal/mol) was stabilized by a hydrogen bond at Ser725 and Ser975 (bonding distance: 2.90 and 3.06 Å, respectively). Like pinocembrin, pinostrobin formed two hydrogen bonds with Ser725 and Ser975 (bonding distance: 2.89 and 3.03 Å, respectively).

## 4. Discussion

In the present study, we investigated the anti-AD potential of three flavonoids from *B. rotunda*, namely cardamonin, pinocembrin, and pinostrobin, by evaluating their capacity to inhibit BACE1. These compounds exhibited powerful inhibitory activities on BACE1 with IC_50_ values in the range of 4.4–28.4 µM. Several other natural BACE1 inhibitors including genistein, biochanin A, daidzein, formononetin, and glycitein had IC_50_ values ranging from at a range of 28->100 µM [27,31]. The results showed that cardamonin, containing an α, β-unsaturated ketone with two aromatic rings, was the most potent BACE1 inhibitor of the three flavonoids. Similar results for the correlation between structure and enzyme inhibition properties were shown in a previous study [32]. Using an antiviral assay, the authors showed that cardamonin exhibited the strongest inhibition of HIV-1 protease activity, with an IC_50_ value of 31.0 μg/mL, compared to pinocembrin (>100 μg/mL) and pinostrobin (>100 μg/mL). Further enzyme kinetic evaluations using various substrates and inhibitor concentrations indicated that all three compounds are noncompetitive BACE1 inhibitors. The docking results indicated that hydrogen bond formation played a key role in the interaction of pinocembrin and pinostrobin with BACE1. However, cardamonin did not form any hydrogen bonds with BACE1 even though it presented the strongest BACE1 inhibition, suggesting that hydrophobic bonds were involved in the interaction between this compound and BACE1. The cardamonin result is in agreement with the report by Castro et al. (2015), which showed that cardamonin inhibited SmATPDase 1 through hydrophobic interactions [33].

Together, the above results indicate that all three compounds are beneficial BACE1 inhibitors, with potential as candidates for use in the prevention and/or treatment of AD. However, few BACE1 inhibitors have been marketed to treat AD due to the difficulty in developing therapeutic agents that can efficiently cross the BBB and reach appropriate concentrations in the cerebral parenchyma, and, moreover, can do so without side effects [34]. Recently, QSAR study approaches have been used to generate predictive models for BBB permeability. To cross the BBB, an active compound must possess the following physicochemical properties: a molecular weight (MW) of less than 450, polar surface area (PSA) of less than 70 Å^2^, and a clog *P* value, used as an assessment of lipophilicity, between 0 and 5 [35]. Cardamonin has a MW of 270.28, a PSA value calculated as 66.76, and a clog *P* of 4.54, obtained by determining the partition coefficient between n-octanol and water [19]. Furthermore, a previous study showed that some synthetic indolyl chalcones could cross the BBB, as determined by a parallel artificial membrane permeability assay [36]. Therefore, cardamonin is predicted to cross the BBB and exert its activity in the brain. An in vitro study by Yang et al. demonstrated that pinocembrin could cross the BBB by passive transport partly through P-glycoprotein [37].

Abnormal phosphorylated tau protein promotes the loss of microtubule-stabilizing ability and may contribute to neuronal degeneration as well as NFT formation [38]. To date, however, it is not known whether our three flavonoids can inhibit tau phosphorylation. However, several studies have revealed aspects in regard to the effect of flavonoids on the tau protein. Myricetin and epicatechin-5-gallate have been shown to suppress heparin-induced tau production and administration of EGCG leads to regulated tau profiles in AD transgenic mouse model, with inhibition of sarkosyl-soluble phosphorylated tau isoforms [39,40]. The authors plan to examine the attenuation effect of the three flavonoids on tau pathology in future studies.

Chronic oral administration of cardamonin (10 mg/kg) for 30 weeks did not elicit any apparent toxicity [41]. Additionally, Charoensin et al. reported that pinostrobin and pinocembrin also did not induce any toxicity or mutagenic effects following oral administration of 100 mg/kg for 7 days in Wister rats, suggesting that all three compounds are safe for consumption [42].

Overall, our tested compounds appear to be safe and potent natural BACE1 inhibitors that hold promise for use in the prevention and/or treatment of AD.

## 5. Conclusions

To the best of our knowledge, the present study is the first to indicate that cardamonin, pinocembrin, and pinostrobin are potent and selective BACE1 inhibitors. In a biological study, cardamonin was six-fold more effective than the two flavanones, pinocembrin and pinostrobin, at inhibiting BACE1. In addition, the inhibition kinetics and docking studies confirmed the allosteric nature of BACE1 inhibition by the tested compounds. Although further in vivo studies of these compounds are required to confirm our present results, our compounds may be good candidates for development as preventative agents against AD through inhibition of Aβ formation.

## Figures and Tables

**Table 1:** Inhibitory activity and kinetic parameters of cardamonin, pinocembrin, and pinostrobin from *B. rotunda* against beta-site amyloid precursor protein cleaving enzyme1 (BACE1).

Compounds	Concentration (µM)	IC_50_ (mean ± SD, µM) ^1^	K_m_	V_max_	K_i_^2^	Inhibition Mode
Cardamonin	0.1	4.35 ± 0.38	833.3	1139	5.1	Noncompetitive
	1			836		
	5			543		
	10			370		
	25			190		
Pinocembrin	1	27.01 ± 2.12	312.5	507	29.3	Noncompetitive
	10			471		
	25			336		
	37.5			270		
	50			229		
Pinostrobin	1	28.44 ± 1.96	116.3	467	30.9	Noncompetitive
	10			362		
	25			268		
	37.5			216		
	50			181		
Resveratrol ^3^		14.59 ± 0.79				

^1^ The IC_50_ values (µM) were calculated from a log dose inhibition curve and expressed as the mean ± SD. ^2^ K_i_ has the dimension of a concentration with the unit of µmol/L (µM). ^3^ Resveratrol was used as a positive reference control in the BACE1.

**Table 2:** Inhibitory activities (%) of cardamonin, pinocembrin, and pinostrobin against α-secretase (tumor necrosis factor-α converting enzyme, TACE) and other serine proteases.

Sample (μM)	TACE	Trypsin	Chymotrypsin	Elastase
Cardamonin				
50	1.38 ± 0.36	1.13 ± 0.18	6.20 ± 0.41	2.29 ± 0.41
100	2.69 ± 1.44	3.74 ± 0.53	4.23 ± 0.55	1.41 ± 0.19
Pinocembrin				
10	5.15 ± 0.98	4.35 ± 0.26	7.18 ± 1.87	3.24 ± 0.19
100	6.95 ± 1.52	4.96 ± 0.27	8.61 ± 1.09	2.56 ± 0.08
Pinostrobin				
10	8.53 ± 1.05	3.19 ± 0.16	3.29 ± 0.25	5.21 ± 0.41
100	9.19 ± 1.20	1.52 ± 0.05	5.41 ± 0.49	2.63 ± 0.37

**Table 3:** Lowest energies and the number of hydrogen bond and hydrophobic interactions of cardamonin, pinocembrin, and pinostrobin with BACE1.

Ligands	Lowest Energy (kcal/mol)	No. of H-Bond	H-Bonds Interacting Residues	Bond Distance (Å)	van der Waals Interacting Residues
Cardamonin	−9.5				Gln12, Leu30, Asp32, Val69, Tyr71, Lys75, Trp76, Phe108, Ile110, Trp115, Gly230
Pinocembrin	−7.9	1	LYS 75	3.09	
Pinostrobin	−7.6	1	PHE 108	1.99	

**Table 4:** Lowest energies and the number of hydrogen and hydrophobic bond interactions of cardamonin, pinocembrin, and pinostrobin with P-glycoprotein (P-gp).

Ligands	Lowest Energy (kcal/mol)	No. of H-Bond	H-Bonds Interacting Residues	Bond distance (Å)	van der Waals Interacting Residues
Cardamonin	−9.78	4	Tyr303, Gln721, Ser725	3.17 2.82 and 3.25 2.96	Phe71, Phe332, Phe724, Phe728, Leu971, Phe974, Ser975, Val978
Pinocembrin	−10.64	2	Ser725, Ser975	2.90 3.06	Phe71, Phe332, Phe724, Phe728, Phe953, Leu971, Phe974, Val978
Pinostrobin	−10.37	2	Ser725,Ser975	2.89 3.03	Phe71, Phe332, Phe724, Phe728, Phe953, Leu971, Phe974, Val978
